# Low spatial frequency surface feature formation during femtosecond laser deep engraving: origins and mechanisms

**DOI:** 10.1038/s41598-026-56143-y

**Published:** 2026-06-03

**Authors:** Evaldas Kažukauskas, Vytautas Jukna, Simas Butkus, Tadas Latvys, Domas Paipulas

**Affiliations:** Laser Research Center, Faculty of Physics, Vilnius University, Saulėtekio Ave. 10, LT-10223 Vilnius, Lithuania

**Keywords:** Engineering, Optics and photonics, Physics

## Abstract

In this study, we explore the formation of high-amplitude, low-spatial-frequency surface features - also commonly referred to as waviness - during laser deep engraving of dielectric materials. This unique phenomenon significantly increases surface roughness and undermines the ability to control it during deep engraving, limiting the technique’s applicability. Here we establish the experimental conditions under which these features are formed and demonstrate how they depend on various processing conditions. Furthermore, we develop a numerical model that considers acoustic wave propagation in a confined cavity. Combining a numerical model with experimental results we demonstrate that the formation of low-spatial-frequency features is governed by the interaction between incident laser pulses and the residual effects of preceding pulses. Finally, building on this insight, we developed strategies to suppress the formation of such surface features, reducing the final surface roughness by a factor of ten.

## Introduction

Femtosecond laser ablation is a non-contact material-removal technique in which material is modified or removed through its interaction with ultrafast laser radiation. Laser ablation offers notable advantages over mechanical methods by eliminating issues related to tool wear, enabling rapid, high-resolution material processing, and remaining compatible with nearly all material classes^1^. The adoption of femtosecond lasers for ablation introduced additional benefits, including reduced heat-affected zones, enhanced control over the modified structure, and others^2^. Over the past decades, femtosecond laser ablation has been implemented for a wide range of applications across fields such as medicine, microfluidics, MEMS, and micro-optics^3–6^. The process of using laser ablation to engrave the material to a precisely defined depth is known as laser engraving^7^. This technique is widely employed in the luxury goods industry for the precision engraving of watches and jewelry^8^, where engraved depths often exceed 100 $\mu$m^9^. Furthermore, deep laser engraving is extensively used in the fabrication of complex, free-form molds^10^ for casting and injection molding, where depths of hundreds of $\mu$m to several mm are required. Besides that, laser engraving serves an important role in shaping and modifying medical components, including orthopedic implants and others^11^.

However, aside from the capability to produce intricate geometries, laser engraving simultaneously affects the surface texture of the material being processed. While deep-engraving, the surface is scanned hundreds of times, resulting in progressive morphological changes. The resulting surface texture determines what functionalities and properties the surface will exhibit. The achieved surface roughness can significantly affect the surface’s optical properties^12^, making it appear glossy or matte, which is of significant importance in the luxury goods industry. Moreover, roughness defines biocompatibility properties of the medical components^13^, thereby affecting the body’s acceptance of laser-engraved implants. Furthermore, deep laser engraving can produce various surface texture artifacts^14^, which may subsequently lead to undesired surface defects on cast or injection-molded components. The provided examples highlight the importance of the ability to control the resulting surface texture during laser deep engraving. Without proper texture management, the engraved structure may fail to perform its intended function and be rendered unusable.

To address this issue, numerous studies have examined how various processing parameters, such as laser fluence, scanning speed, burst regime, and pulse overlap affect the final surface roughness during laser engraving^15–18^. Further insights were gained from studies examining how various scanning strategies impact final surface texture. The surface scanning approaches that included interlacing^19^, radial scanning^20^, power gradient method^21^, rotation of the layers^22^, and the displacement of the layers^14^ have been tested on various materials to evaluate their respective advantages and limitations in controlling surface texture during laser engraving. Contributing to this line of work in our previous study^23^ we investigated the evolution of fused silica surface texture during laser deep engraving by considering changes in the surface spatial spectra. It was demonstrated that during laser deep engraving (depth > 500 $\mu$m), the unforeseen growth in surface roughness is observed that cannot be attributed to algorithm-related effects. It was revealed that the observed roughness increase is associated with the formation of high-amplitude, low-spatial-frequency features (HALSFF), corresponding to the generation of new waviness components within the surface texture. The generated low-frequency components exhibited a high amplitude of PV (peak-to-valley) > 3 $\mu$m, significantly increasing overall surface roughness. The mechanisms behind the formation of these spatial features remained unclear, leaving the question unresolved. However, the uncontrollable increase in surface roughness during laser deep engraving limits precise control over surface texture, making the resulting surfaces unsuitable for certain applications.

Therefore, this study conducts an in-depth investigation regarding the formation of new, high-amplitude, low-spatial-frequency features during laser deep engraving, with the aim of understanding their origins and identifying strategies to eliminate them. In the course of the study, we establish the conditions that lead to the generation of the aforementioned surface features and investigate how the generated structures vary in shape and amplitude depending on different processing conditions. The results indicate that the origins of the generated surface features lie in the interplay between the propagating acoustic waves and the incident laser pulses. To test the hypothesis, we developed a numerical model that considers the propagation of acoustic waves generated during laser processing and compared the numerical findings with experimental observations. Finally, based on the gained knowledge, we propose strategies to prevent the formation of HALSFF during deep laser engraving, thereby significantly enhancing control over surface texture.

## Results

### Experimental results

**Fig. 1:**
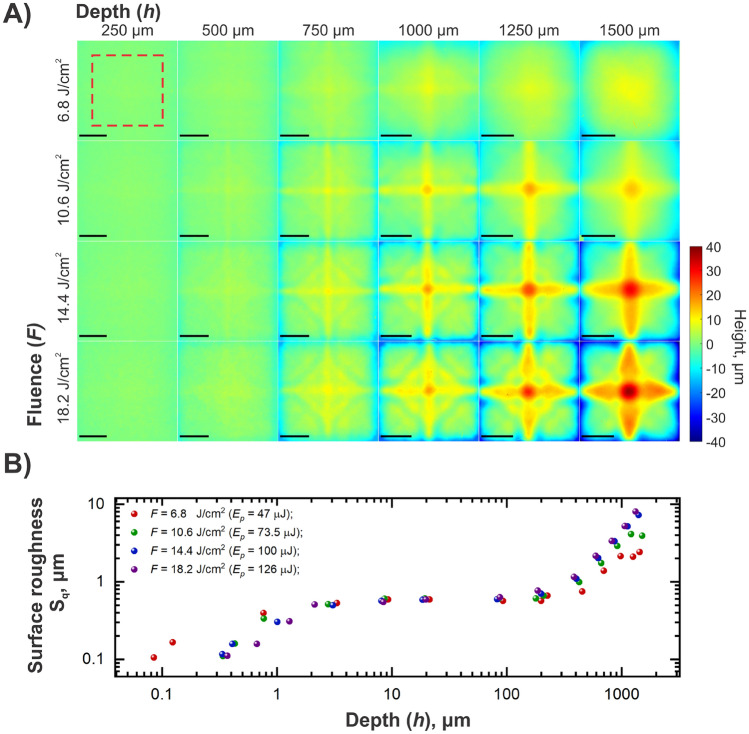
Height maps of the bottom surfaces of deep ablated rectangular cavities of varying depths produced using different fluences *F* (A). Surface roughness parameter $S_q$ dependence on the engraved depth, when different *F* values are used (B). Other laser parameters were fixed: scanning speed *v* = 2325 mm/s, scanning pitch $dy=dx= 23.3$ $\mu$m. The black bar indicates a length of 1 mm, the red dashed rectangle shows the area from which the roughness was calculated (representing 2.1 mm x 2.1 mm in size).

**Fig. 2:**
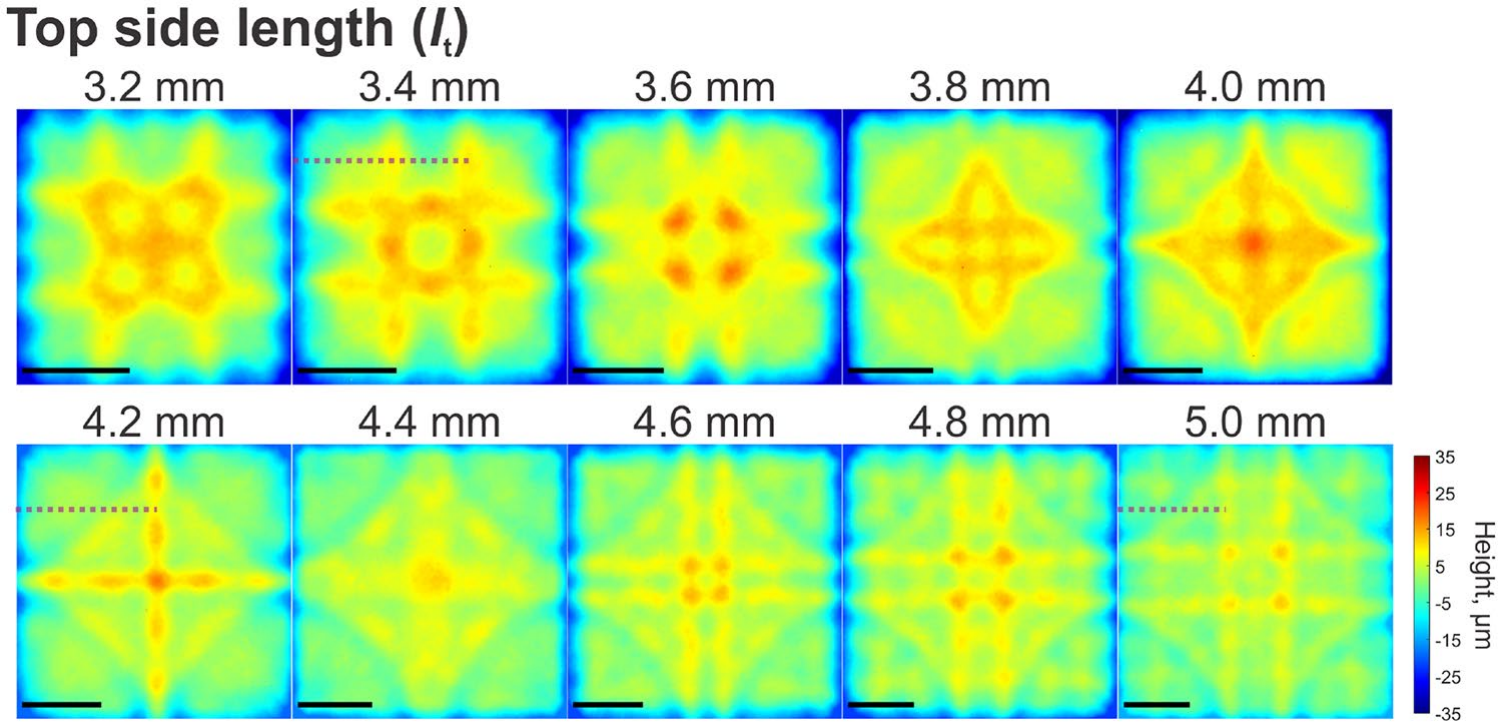
Height maps of the bottom surfaces of deep ablated rectangular cavities with top side lengths of $l_t$. Laser processing parameters used: fluence *F* = 18.2 $~\text {J/cm}^2$, scanning speed *v* = 2325 mm/s, scanning pitch $dy=dx= 23.3$ $\mu$m, number of scans *N* = 775, engraved depth *h* = 820 $\mu$m. The black bar indicates a length of 1 mm. The dashed purple line indicates the length of 1.78 mm.

To establish the conditions under which HASLFF are formed, a systematic investigation of their geometric constrains dependence on various processing parameters was conducted. We first examined how the structural characteristics of the formed HALSFF depend on the laser fluence values used during the processing. The fluence was varied by changing the pulse energy ($E_p$) and is expressed as: $F=2E_p/(\pi \omega _0^2)$. The experiment consisted of engraving rectangular cavities with dimensions of 4 $\times$ 4 mm to a specified depth using different fluence values. To ensure the same depth was reached while using different *F* values, the number of scans *N* was varied according to the change in ablation rate. The resulting bottom surfaces of the ablated cavities are presented in Fig. 1, part A. As can be seen, when deep ablating 4x4 mm size cavities, HALSFF exhibiting cross-like shapes are formed. The patterns begin to surface when the engraved depth of $h \ge$ 500 $\mu m$ is reached. The measured surface roughness $S_q$ values, given in part B of Fig. 1, confirm that the formation of HALSFF is responsible for the increase in surface roughness observed after prolonged engraving, as was suggested in our previous work^23^. It is observed that the formed surface features are ablation depth *h* and fluence *F* dependent. Once surfaced, their peak-to-valley (PV) values, defined as difference in height between base level and induced surface feature peak value, increase with engraved depth *h*, leading to a corresponding rise in surface roughness. The rate of increase in $S_q$ is governed by the fluence, with higher *F* values producing a more rapid growth of surface roughness. At a Fluence value of $18.2~\text {J/cm}^2$, the surface roughness $S_q$ reaches $8~\mu \text {m}$ at an engraved depth of approximately $1500~\mu \text {m}$, whereas at $6.8~\text {J/cm}^2$ the roughness is only $2.4~\mu \text {m}$. Accordingly, at $F = 18.2~\text {J/cm}^2$ the height maps reveal pronounced HALSFF with well-defined shapes of high PV values, whereas at $F = 6.8~\text {J/cm}^2$ these features are barely discernible. It is plausible that at even lower fluence values ($F \le 6.8~\text {J/cm}^2$) HALSFF formation could become negligible, indicating that fluence is one of the critical factors in the formation of these surface features. It is further observed that in addition to the increase of PV and $S_q$ values, the features shape gradually changes and broadens as engraving progresses. It suggests that this phenomenon may be cavity size dependent, as with increasing depth of the cavity, the bottom surface area narrows due to the sidewalls being non-perpendicular. Therefore, the subsequent analysis focused on investigating the dependence of HALSFF formation on the ablated cavity size. The top side length of the ablated rectangular cavities was varied from 3.2 mm to 5 mm, while keeping the laser processing parameters constant. The resultant morphologies of the bottom surfaces of deep ablated cavities are shown in Fig. 2.

As can be observed in the figure, HALSFF were formed in all cases; however, the resulting surface features exhibit different patterns depending on the side length of the ablated cavity. The patterns, despite their different shapes, show signs of rectangular geometry, suggesting that the contour of the cavity plays an important role in their formation. Furthermore, a more detailed analysis of the pattern shapes revealed the presence of a characteristic fixed distance of 1.78 mm (displayed as dashed purple line in the figure) that is common to all patterns. For a cavity with a side length of 4.2 mm, the distance from the sidewall to the cross maximum is approximately 1.78 mm. For a cavity with a side length of 5 mm, the same distance (1.78 mm) is observed between the sidewall and the first major parallel ridge. Similarly, for a cavity with a side length of 3.4 mm, a distance of 1.78 mm is measured between the sidewall and the second major parallel ridge. A certain evolution of the pattern shape with decreasing side length *l* can be observed. First, when the cavity side length is 5 mm, the pattern exhibits four major ridges-two horizontal and two vertical-each located 1.78 mm from the sidewalls. As the cavity size decreases, the distance between the ridges and the sidewall remains constant, causing the ridges to move closer together until they merge at a cavity side length of 4.2 mm. With a further decrease in cavity size, the ridges split again, this time maintaining a fixed distance of 1.78 mm between the sidewall and the second ridge. The characteristic evolution of the HALSFF shapes, together with the consistent fixed distance observed in all patterns, suggests that the shape of the formed surface features is determined by the cavity size and shape.

**Fig. 3:**
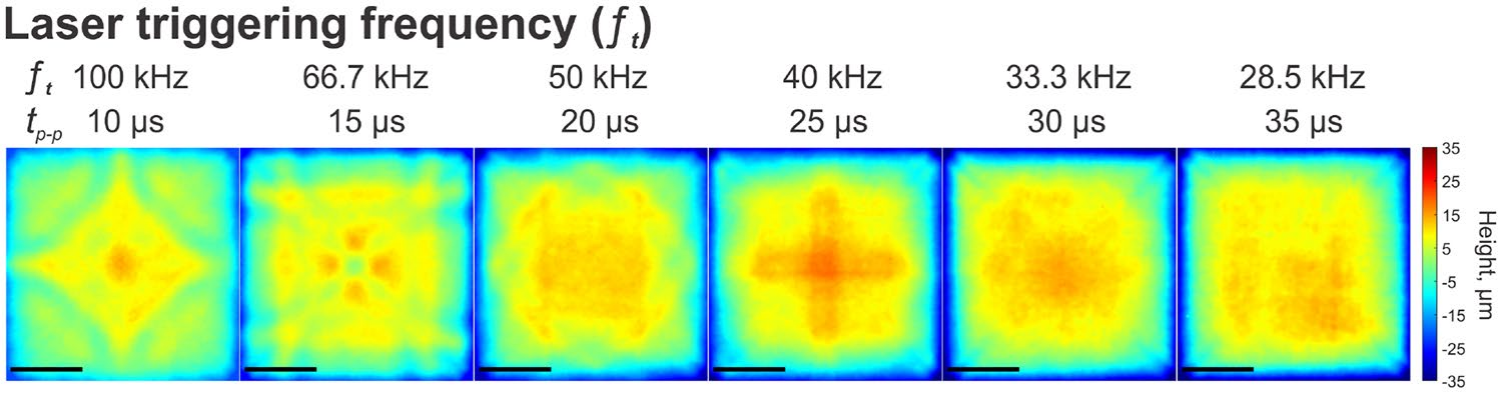
Height maps of the bottom surfaces of deep ablated rectangular cavities, when scanning is performed under identical conditions, with only varying parameter being the temporal duration between consecutive laser pulses $t_{p-p}$. Laser processing parameters used: fluence *F* = 18.2 $~\text {J/cm}^2$, overlap of the craters $\Omega$ = 20 $\%$, number of scans *N* = 775, engraved depth *h* = 820 $\mu$m. The black bar indicates a length of 1 mm.

Next, an investigation was conducted to examine the influence of temporal and spatial scanning parameters on the formation of HALSFF. The temporal scanning parameter here is defined as the time interval between consecutive laser pulses. It was varied by changing the laser repetition rate $f_t$ with a pulse picker and accordingly adjusting the scanning speed to maintain a constant pulse pitch *dx* between laser pulses. As a result, the surfaces were scanned under identical conditions-maintaining the pulse pitch *dx*, line pitch *dy*, pulse energy $E_p$, scanning strategy, and number of passes *N*, with only varying parameter being the time interval between consecutive pulses $t_{p-p}$. Surfaces formed with different $t_{p-p}$ values are displayed in the Fig. 3.

The results show that diverse $t_{p-p}$ values lead to the formation of different HALSFF patterns, while the PV value of the structures remains mostly preserved. Furthermore, it is seen that when the temporal separation between pulses is $t_{p-p} \ge$ 30 $\mu s$ ($f_t = 33.3$ kHz), no distinguishable HALSFF are observed, with the only remaining low spatial frequency surface feature being the convex surface. The formation of a convex surface during multilayer laser ablation using high pulse energy laser pulses is a well-known phenomenon and is documented in the literature^24^. It is attributed to the partial reflection of incident laser pulses by non-perpendicular sidewalls toward the bottom surface near the sidewalls, which enhances local ablation and eventually results in trench formation. The absence of HALSFF at large temporal separations between laser pulses indicates that the physical mechanism underlying HALSFF formation relies on a high laser repetition rate. It was demonstrated, that HALSFF formation can be suppressed by performing laser processing at $f_t \le$ 30 kHz; however, this approach is impractical, as it significantly prolongs the processing duration. For instance, ablating a $4 \times 4 \,\text {mm}^2$ area cavity to a depth of $820 \,\mu \text {m}$ required 10 minutes (corresponding to 775 scans) at $f_r = 100 \,\text {kHz}$, whereas at $f_r = 33.3 \,\text {kHz}$, the process took 25 minutes, resulting in approximately 2.5-fold increase in duration.

**Fig. 4:**
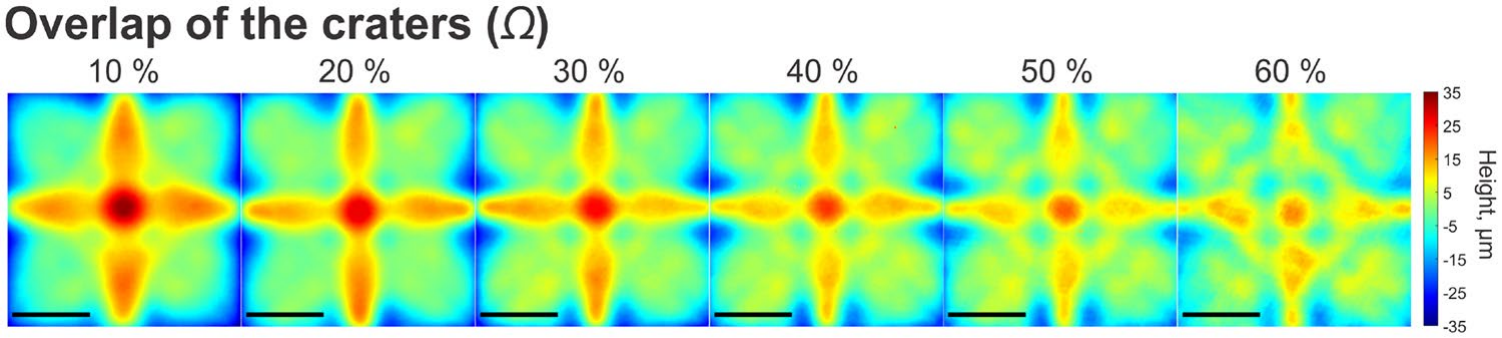
Height maps of the bottom surfaces of deep ablated rectangular cavities obtained using different crater overlap values $\Omega$. Laser processing parameters used: fluence *F* = 18.2 $~\text {J/cm}^2$, scanning speed *v*, line pitch *dy* and number of scans *N* were varied to reach an engraved depth of *h* $\approx$ 1000 $\mu$m. The black bar indicates a length of 1 mm.

Subsequently, the influence of spatial scanning parameters on HALSFF formation was examined. In this case, the temporal separation between laser pulses was kept constant, corresponding to a fixed laser repetition rate of 100 kHz, while the cavities were engraved using different overlap of the craters ($\Omega$) values. The overlaps in both the *x* and *y* directions ($\Omega _x$ and $\Omega _y$) were varied simultaneously to ensure uniform ablation. It should be noted that number of scans *N* was adjusted for each $\Omega$ value to ensure the same engraved depth of *h* $\approx$ 1000 $\mu$m is achieved. The obtained surfaces after engraving with different overalp of the crater values are shown in Fig. 4. The morphologies indicate that the pattern of generated HALSFF is independent of the spatial scanning parameters. Across all cases, the pattern remains essentially the same, while the PV values show minor variations. These can be attributed to the fact that the number of scans *N* applied for different $\Omega$ values was not equal. The number of scans required to reach a 1 mm engraved depth was 224 for a crater overlap of $\Omega = 60\%$, compared to 1428 scans for $\Omega = 10\%$, corresponding to more than a sixfold increase. It has been previously shown that HALSFF formation is a cumulative process, with the PV value increasing as the number of layers grows. This characteristic of the HALSFF formation mechanism explains well why the PV value of the pattern generated at $\Omega = 10\%$ exceeds that obtained at $\Omega = 60\%$. Moreover, a closer examination of the figures reveals that as $\Omega$ increases, not only does the PV value of the pattern change, but the pattern itself becomes increasingly distorted. The distortion at high $\Omega$ values is caused by the increased surface roughness at higher spatial frequencies. It was demonstrated in the literature^25^, that when ablating brittle dielectric materials, such as fused silica, the optimal crater overlap for minimal surface roughness is $\Omega \approx 20$ $\%$, any further increase or decrease in overlap beyond this value leads to higher surface roughness. This is exactly what is observed in the Fig. 4, at a low overlap value of $\Omega = 10\%$, the HALSFF surface is smooth, whereas at $\Omega = 60\%$, the same HALSFF is observed; however, this time its surface roughness is much higher, giving the impression of the form distortion. Taken together, although spatial scanning parameters influence surface roughness, they do not affect HALSFF formation; these surface features form independently of the scanning speed or scanning pitch used.

### Numerical model for acoustic wave propagation

During laser ablation, each incident laser pulse, in addition to material removal, also generates a pressure disturbance in the surrounding environment^26^. This disturbance initially propagates as a shock wave and rapidly relaxes into an acoustic wave that continues to travel across the ablation region. Because these acoustic waves persist much longer than the typical inter-pulse separation in high-repetition-rate laser systems, they remain present when subsequent pulses arrive. In this work, we propose that the interaction between consecutive pulses and the residual pressure field generated by earlier pulses plays a defining role in the formation of HALSFF observed in our experiments.

To examine this hypothesis, we developed a numerical model to estimate the acoustic field distribution over the processing area at different time delays after the initial laser-matter interaction. Rather than simulating the full thermo-mechanical dynamics of the material, our model focuses specifically on the propagation of the pulse-generated pressure wave through the surrounding air and its reflections from the ablated cavity boundaries. The complete acoustic field dynamic would ordinarily be governed by the pressure-wave equation:

1 $$\begin{aligned} \nabla ^2 p-\frac{1}{v_0^2}\frac{\partial ^2 p}{\partial ^2 t}=0, \end{aligned}$$

where *p* is the acoustic pressure and $v_0$ is the speed of sound. Solving this equation directly across the entire ablation domain, including multiple reflections, is computationally prohibitive for the timescales of interest; therefore, appropriate approximations were introduced. Each laser pulse was approximated as a point-like emitter of a spherical pressure wave originating from the laser-matter interaction site. Reflections from ablated cavity walls were incorporated using the method of images^27^, where each planar boundary is substituted by a virtual source placed symmetrically across the boundary plane. Due to very large differences in impedance between the air ($Z_\text {air} \approx 4.1 \times 10^2$ Rayls) and fused silica ($Z_\text {FS} \approx 3 \times 10^7$ Rayls), almost all of the acoustic wave energy is reflected ($R=\frac{Z_{FS}-Z_{air}}{Z_{FS}+Z_{air}}\approx 1$). Therefore, the virtual sources were assigned the same amplitudes as the original sources. This simplified geometric-acoustics description captures the essential reflection and interference behaviors without resorting to computationally expensive numerical integration of the full wave equation 1. The temporal evolution of the pressure at each point was assumed to follow a modified Friedlander waveform^28^, which characterizes the typical pressure dynamics of laser-induced shock waves in the air, featuring a rapid rise to peak overpressure, followed by an exponential decay and a subsequent negative rarefaction phase^29^. To account for a finite rising time of the pressure wave, an additional, second exponential term was introduced. The resulting pressure-time dependence is expressed as:

2 $$\begin{aligned} P(t)=P_{max}C\left( 1-\frac{(t+t_0)}{\tau _p}\right) exp\left( -\frac{(t+t_0)}{\tau _p}\right) \left( 1-exp\left( -\frac{(t+t_0)}{\tau _r}\right) \right) , \end{aligned}$$

here, $P_{max}$ denotes the peak pressure, while *C* and $t_0$ are numerically determined normalization constants, with $t_0$ defining the temporal location of the pressure peak. $\tau _p$ is the characteristic decay time (positive-phase duration) and $\tau _r$ is the rise time. For the present calculations, the characteristic values of $\tau _p=1$ $\mu$s, $\tau _r=0.25$ $\mu$s were chosen according to the literature^29^. To account for spherical spreading, the pressure amplitude at a distance *r* from the source is given by $P(r,t)=P(t)/r$.

**Fig. 5:**
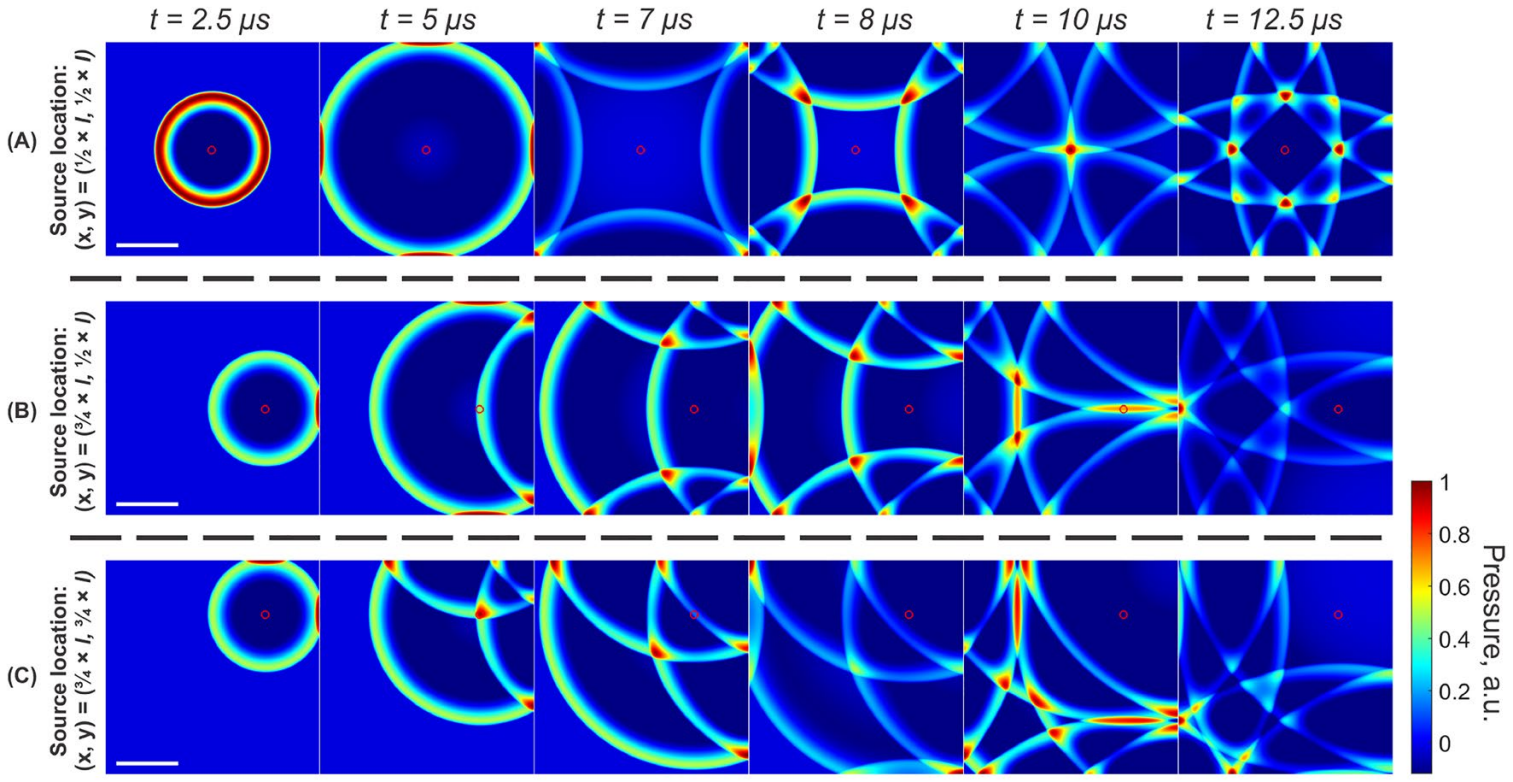
Pressure distributions of a propagating acoustic wave at different time delays (2.5; 5; 7; 8; 10; 12.5 $\mu$s) after initiation. Parts A, B, and C illustrate scenarios with a single source located at different positions (indicated by a red circle). Amplitudes in each image are normalized. The scale bar represents 1 mm in length and variable *l* stands for a side length of a rectangular cavity.

The numerical model was used to compute acoustic field distributions at various time delays after the initial laser-matter interaction. The rectangular cavity was assigned a length $l = 3.43\ \text {mm}$ (to be comparable with experimental conditions), and the speed of sound in air was set to $v_0 = 343\ \text {m/s}$. The computed pressure distributions are displayed in Fig. 5, with different parts of the figure corresponding to scenarios where the acoustic wave source is positioned at different locations on the surface. Part (A) corresponds to the source located at the center of the processing area ($x = \frac{1}{2}l$, $y = \frac{1}{2}l$), part (B) to a source shifted laterally ($x = \frac{3}{4}l$, $y = \frac{1}{2}l$), and part (C) to a source shifted diagonally ($x = \frac{3}{4}l$, $y = \frac{3}{4}l$). In all three parts, the evolutions of an acoustic fields are observed, highlighting the emergence of complex field distributions resulting from multiple reflections and interference effects. Upon reflection, the fields develop localized overpressure regions, whose positions are governed by the cavity’s size, geometry and the initial source location. As observed, fields with strong localized overpressure regions can persist for extended durations ($t> 10$ $\mu$s), lasting until the arrival of a subsequent laser pulse on the surface. This opens the possibility for interaction between a subsequent laser pulse and the overpressure regions, provided their locations coincide.

## Discussion

**Fig. 6:**
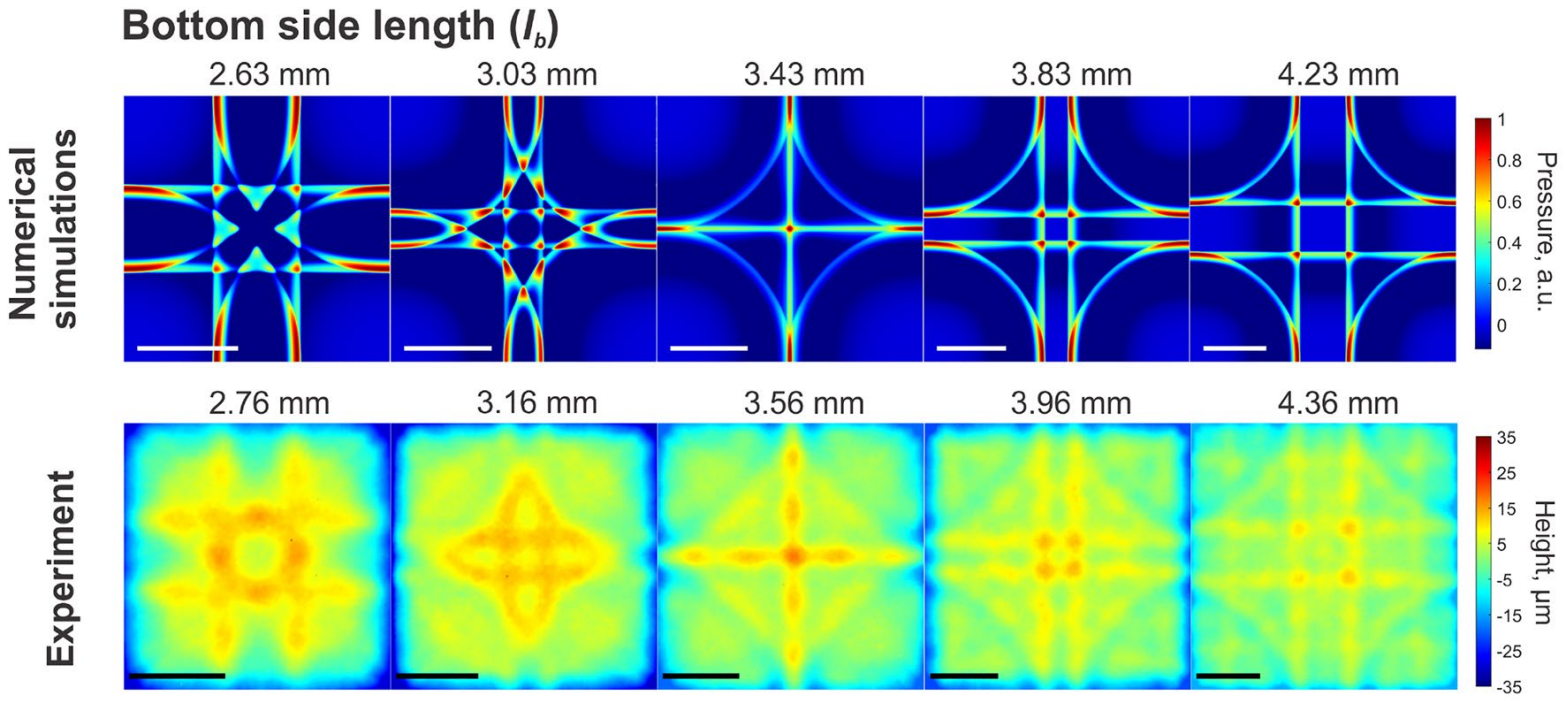
Pressure maps of surfaces with different bottom side lengths of $l_b$ generated using numerical model presented in the study (top row). Height maps of the bottom surfaces of deep ablated rectangular cavities with bottom side lengths of $l_b$ (bottom row). Laser processing parameters used in the experiments are detailed in Fig. 2 caption.

The experimental results presented earlier provide valuable insights into the conditions necessary for HALSFF formation; however, they are insufficient on their own to elucidate the underlying physical mechanisms governing this process. Observations show that HALSFF formation requires short temporal separation between consecutive pulses, the presence of the sidewalls, and high fluence values, suggesting the involvement of both the laser pulse and residual laser-induced phenomena behind the formation of these features. To examine that, the acoustic fields generated during the laser processing were studied using the developed numerical model. An imaginary plane was constructed and populated with multiple spherical wave sources imitating laser scanning. Using the numerical model, the propagation and reflection of each spherical wave within the ablated cavity were traced over the temporal delay between two laser pulses, recording the final positions of the wavefronts. By superposing the contributions from all spherical wave sources, the final pressure map could be obtained, showing the residual pressure amplitude distribution over the ablated cavity at the moment the second laser pulse arrives. The calculated pressure maps for different size cavities are displayed in Fig. 6, top row. The bottom row of the figure shows the experimentally generated HALSFF during laser deep engraving of similarly sized cavities.

**Fig. 7:**
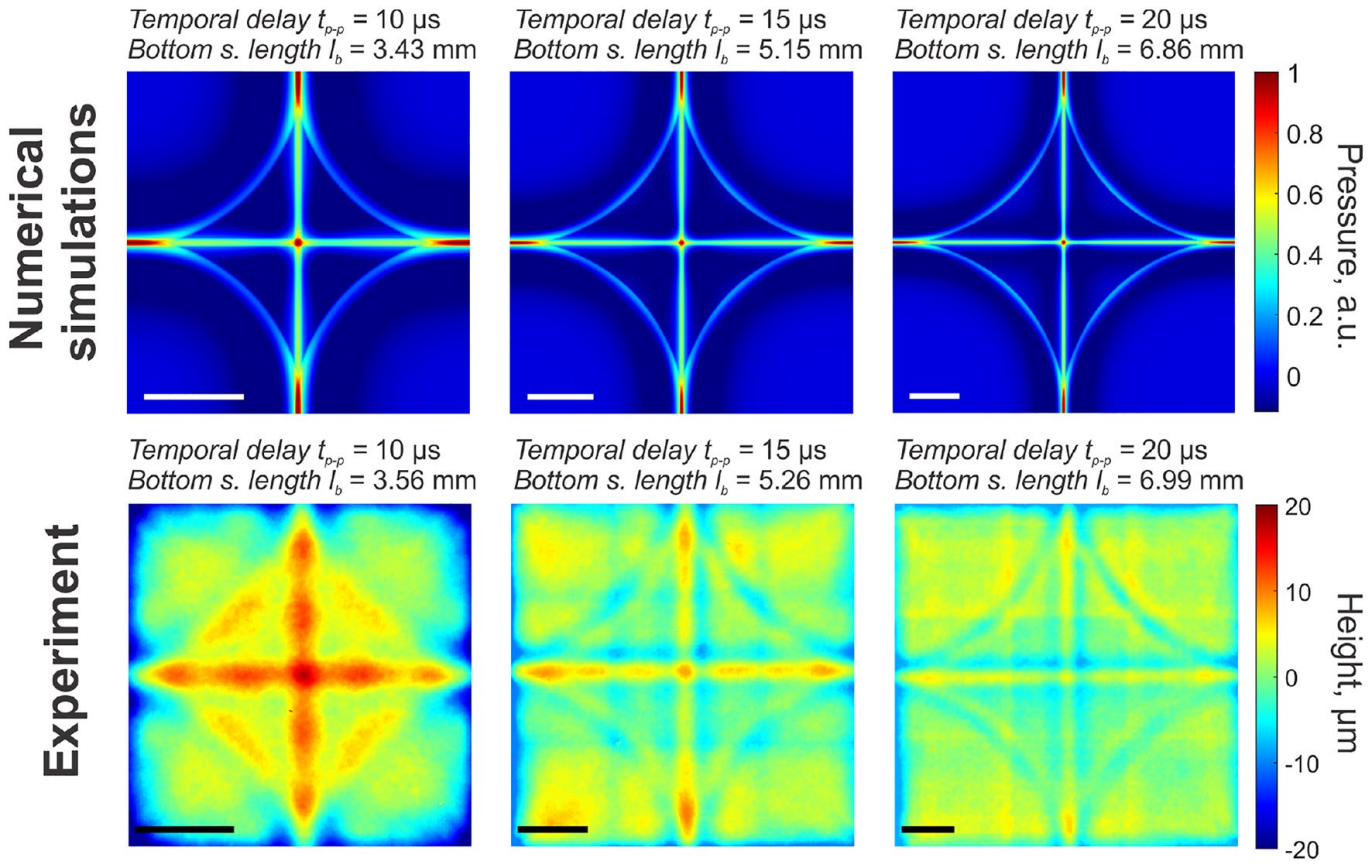
Pressure maps of surfaces with different bottom side lengths of $l_b$ and temporal delay $t_{p-p}$ values generated using the numerical model presented in the study (top row). Height maps of the bottom surfaces of deep ablated rectangular cavities with bottom side lengths of $l_b$ and temporal delay values of $t_{p-p}$ (bottom row). Laser processing parameters used: fluence *F* = 18.2 $~\text {J/cm}^2$, overlap of the craters $\Omega$ = 20 $\%$, number of scans *N* = 775, engraved depth *h* = 820 $\mu$m.

Comparison of the pressure maps with the formed HALSFF reveals a clear similarity in their pattern shapes. It can be observed that regions of highest pressure correspond to protrusions in the HALSFF, whereas areas of minimal pressure exhibit a relatively homogeneous surface. This suggests that during laser processing, a laser pulse passing directly through the wavefront of a propagating acoustic wave generated by a preceding pulse results in the formation of a protrusion. Reports in the literature^30^ show that the initial pressure of the shock wave generated during femtosecond laser–matter interaction can reach GPa levels. After traveling a distance of 4 mm (in line with experimental observations), its pressure is reduced to the range of 10 kPa to 1 MPa, following an approximate $\sim$ $r_0/r$ scaling. Applying the simplified relation of refractive index change to pressure change, $\Delta n \approx (n_0-1)\Delta p/p$, the estimated pressure variations of traveled acoustic wave correspond to a refractive index change of $\Delta n \approx$ 10$^{-5}$ to 10$^{-3}$. Such refractive index variations are sufficient to introduce local phase distortions in the propagating laser beam, leading to degraded focusing and consequently reduced ablation rate. The experimentally estimated decrease in ablation rate was found to be $\delta R_t \le$ 5%. While resultant local depth variations are negligible for a single scan, the cumulative buildup over multiple passes ($N>$ 100) becomes significant, leading to the formation of high-amplitude, low-spatial-frequency features. This theory aligns well with the observation that HALSFF formation occur only under deep engraving conditions, when the cavity sidewalls are established, as they are necessary for confining the acoustic waves within the processing cavity. Furthermore, the analysis of experimental results have shown that higher fluence values ($F \ge 6.8~\text {J/cm}^2$) are required for pronounced HALSFF formation. This can now be explained by considering that the pressure of the generated shock wave is directly proportional to the pulse energy applied^31^. As a result, higher pulse energies (fluence values) produce stronger perturbations for subsequent laser pulses, facilitating HASLFF formation. However, while the Fig. 6 offers a compelling explanation of HALSFF formation, it also reveals certain discrepancies between the model and the experimental results. As shown in the figure, the cavity lengths required to produce identical HALSFF patterns differ between the model and the experiment by up to 5 $\%$. Furthermore, although the main patterns coincide in both the model and the experiment, the experimentally generated surfaces exhibit additional small-scale features surrounding the main pattern that are not captured in the modeling results. These discrepancies between the model and the experiment are believed to arise from additional factors not accounted for in the model, such as non-perpendicular side walls, textured side walls, variations in airborne acoustic wave speed due to surface heating, and higher-order reflections (second, third, etc.) that may also influence the results. Nevertheless, the model provides a comprehensive explanation of the governing physical mechanisms and enables the prediction of the main HALSFF pattern formed during multilayer laser engraving. To further validate the proposed physical mechanism underlying HALSFF formation, an attempt was made to reproduce identical HALSFF patterns under different processing conditions. It was observed in Fig. 3 that different temporal delays between laser pulses $t_{p-p}$ yield different HALSFF patterns, as longer delays allow the acoustic wave to propagate extended distances, thereby perturbing the laser processing at different locations. Using the presented numerical model, the cavity side lengths required to generate the same pattern for different temporal delays between laser pulses $t_{p-p}$ were calculated. The generated HALSFF patterns under different temporal delay $t_{p-p}$ and side lengths *l* values are shown in Fig. 7.

The top row shows numerical simulation results, while bottom row - experimental results. As can be seen, identical cross like pattern was formed in all the cases, showcasing the accuracy of the model and validating proposed physical mechanism governing the formation of HALSFF. Furthermore, it is observed that when the cavity’s length is large ($l_b =$ 6.99 mm), the HALSFF PV value is much smaller compared to the case when the cavity’s length is small ($l_b =$ 3.56 mm). This effect is caused by the pressure loss of the acoustic wavefront due to its expansion during propagation, since the amplitude of a spherical wave decreases with distance according to $A \propto 1/r$, where *r* is the distance traveled. On top of that, when processing larger areas, due to longer temporal delays between successive scanned lines, the heat accumulation is less pronounced, eliminating the influence of variable sound speed in heated media and other morphological phenomena that might occur under increased temperature conditions. The eliminated additional factors, result in the better agreement between model and the experiment, as seen in the case where $l_b \approx$ 7 mm.

**Fig. 8:**
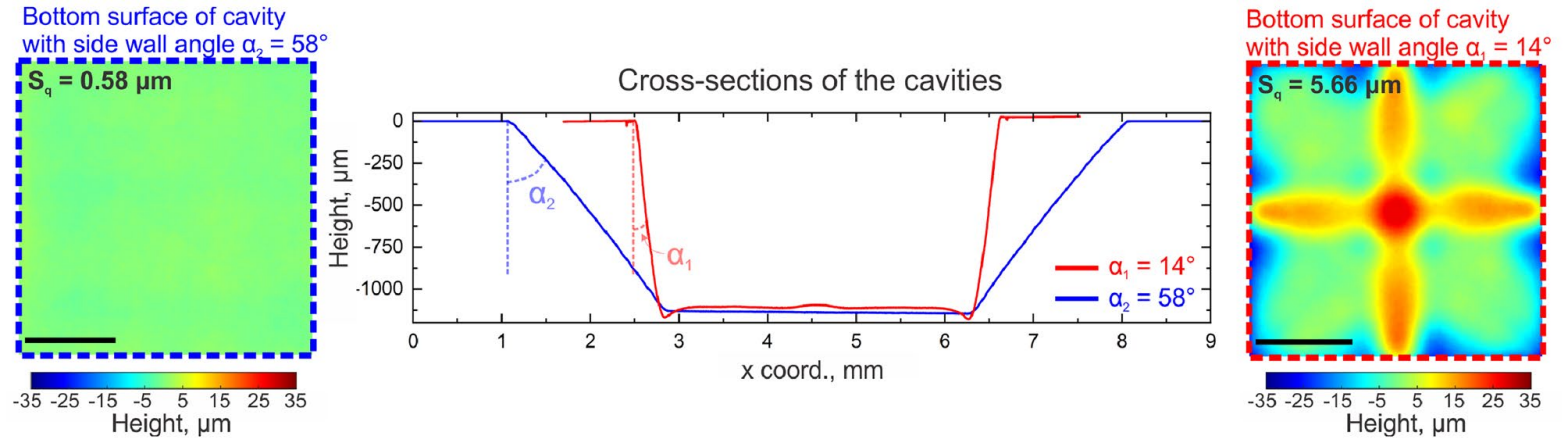
Cross-sections of the cavities with sidewalls of different angles (in the middle). eight maps of the bottom surfaces of deep ablated rectangular cavities with sidewalls of different angles (on the sides). Laser processing parameters used in both cases: fluence *F* = 18.2 $~\text {J/cm}^2$, overlap of the craters $\Omega$ = 20 $\%$, number of scans *N* = 1111, engraved depth *h* = 1130 $\mu$m. The black bar indicates a length of 1 mm. See in text for explanation on how different side-wall angles were achieved.

Finally, because the formation of HALSFF is an undesirable effect that leads to an uncontrollable increase in surface roughness during prolonged laser engraving, we propose several strategies to prevent its occurrence. One way to prevent the formation of HALSFF has already been demonstrated and relies on increasing temporal separation between consecutive laser pulses. Increasing the temporal separation between laser pulses causes the spherical wave to propagate over a longer distance, resulting in a greater loss of wavefront pressure. If the propagation distance is sufficiently long, the wavefront pressure of the spherical wave becomes too weak to perturb the laser pulse, preventing a decrease in ablation efficiency. Furthermore, extended acoustic wave travel increases the number of reflections and may even cause the wave to exit the processing area. The results in Fig. 3 disclosed that $t_{p-p} \ge$ 30 $\mu$s (corresponding to $f_t = 33.3$ kHz) was necessary for the HALSFF to be barely distinguishable, suggesting that such duration of acoustic wave travel was sufficient for the wave to esacpe the cavity or loose its pressure to a negligible level. However, although this solution prevents the formation of HALSFF, it significantly prolongs the processing duration ($\ge$ 2.5 times, as demonstrated previously), making it ineffective. Another, more efficient approach in preventing the formation of these features relies on controlling the propagation direction of the spherical acoustic waves. It was observed that for the patterns to form, the presence of the steep sidewalls is necessary. Since the angle of reflection is identical to the angle of incidence^32^ (in the consideration of ray optics), the cavity side-wall angle determines the pointing vector of the reflected acoustic spherical wave. Under typical processing conditions, the sidewalls with an angle of $\alpha \approx$ $10^\circ$ are formed, where $\alpha$ is the angle between the sidewall and the vertical. Such steep side-walls reflect the spherical waves back over the processing area, creating conditions for them to interact with subsequent laser pulses and perturb them. However, if a side-wall angle $\alpha \ge 45^\circ$ would be achieved, the spherical waves would be reflected outside the cavity, eliminating their perturbation of the subsequent laser pulses. In order to test it, the cavity with side-wall angle of $\alpha \approx$ $60^\circ$ was formed. This was achieved by initiating the ablation process with increased cavity side length $l_t$ and reducing the side length of each subsequent layer by a constant amount of $\delta l_t$, thereby forming a cavity with identical depth and bottom side length $l_b$ as before, but with different side-wall angle. The results are shown in Fig. 8.

From a comparison of the height maps of the bottom surfaces of cavities with different side-wall angles, it is evident that a high side-wall angle $\alpha =$ $58^\circ$ prevents the formation of HALSFF, thereby confirming the previously proposed hypothesis. Furthermore, in addition to preventing the formation of HALSFF, a large side-wall angle also suppresses the formation of trenches around the perimeter of the bottom surface near the side-walls. The introduced benefits of large side-wall angle resulted in the total reduction of the bottom surface roughness by ten-fold, from $S_q$ = 5.66 $\mu$m when $\alpha = 14^\circ$ was used to $S_q$ = 0.58 $\mu$m at $\alpha$ value of $58^\circ$. However, producing cavities with shallow sidewalls requires removing more of the material, thereby extending the processing duration. The ablation of a $4 \times 4 \,\text {mm}^2$ cavity with sidewalls of $14^\circ$ to a depth of $1130 \,\mu \text {m}$ required 14 minutes, whereas producing an identical cavity with sidewalls of $58^\circ$ took 24 minutes, corresponding to a 1.7-fold increase in processing time. Compared to mitigation strategy based on reducing the laser repetition rate, which prolonged processing duration by 2.5 times, this approach comes superior, offering a more effective solution for mitigating HALSFF formation. In addition to the solutions presented, other plausible approaches for suppressing HALSFF formation exist, such as performing laser processing in a vacuum, applying a high-pressure gas stream or working in a burst regime. However, these methods were not tested in the framework of this study, and their effectiveness therefore remains unknown.

## Conclusions

In this study an in-depth investigation was carried out to reveal the governing physical mechanisms behind the formation of parasitic, high amplitude low spatial frequency features (HALSFF) that are formed during laser deep engraving. The experimental results and numerical calculations revealed that HALSFF are formed due to the laser pulse generated acoustic wave perturbation of the consecutive laser pulse wavefront, which results in the reduced local ablation rate. Each laser pulse generates a shock wave that upon expansion transforms into an acoustic wave, continuing its propagation further over the processing area. If the subsequent incident laser pulse overlaps with the propagating acoustic wave, its wavefront is perturbed, resulting in reduced ablation efficiency at incident location. The cavity size and form determines the locations where this effect takes place, thereby defining the pattern of formed HALSFF. The effect was only observed when fluence values of $F \ge 6.8~\text {J/cm}^2$ were used, as such a magnitude of fluence was required to generate sufficiently strong acoustic waves capable of inducing perturbations. Furthermore, this effect occurs only in the presence of steep side-walls, which confine the propagating acoustic waves over the processing area. It was demonstrated that the formation of HASLFF can be suppressed by increasing the temporal delay between consecutive laser pulses. At longer delays (in our case, $t_{p-p} \ge 30~\mu$s, achieved at $f_t = 33.3$ kHz), the laser-induced acoustic wave undergoes sufficient amplitude attenuation such that it can no longer perturb the subsequent laser pulses or may even exit the processing area due to multiple reflections. Another suggested approach in omitting HALSFF formation includes forming cavities with sidewall angles of $\alpha> 45^\circ$. Under these conditions, the laser-induced acoustic waves, after reflecting from the sidewalls, are directed to exit the processing region, thereby eliminating their interaction with subsequent laser pulses. It was shown that suppression of HALSFF formation can reduce the surface roughness by an order of magnitude, with $S_q$ decreasing from 5.66 $\mu$m to 0.58 $\mu$m during laser deep engraving to a depth of 1 mm. The physical mechanism underlying HALSFF formation identified in this study constitutes a novel contribution to the field of laser micromachining. This discovery enables precise control over surface roughness during laser deep engraving, thereby facilitating its application in areas such as well-textured mold fabrication and high-precision processing of luxury goods.

## Methods

To identify the conditions that result in the formation of high-amplitude, low-spatial-frequency features, laser processing experiments were performed on fused silica samples using a femtosecond Yb:KGW laser (“Carbide”, manufactured by Light Conversion, UAB). The laser could deliver a maximum of 40 W of laser power with a pulse duration of 210 fs (measured at FWHM). The laser was capable of operating at different repetition rates up to 2 MHz, emitting fundamental ($\lambda$ = 1030 nm) laser radiation. The polarization state of the laser radiation was maintained linear. The laser power was controlled using an external attenuator that was composed of $\lambda$/2 waveplate and a Brewster-type polarizer. The laser beam was guided using high reflectivity dielectric mirrors, and the scanning was performed using a galvanometer scanner (“IntelliSCAN10se”, Scanlab GmbH) in combination with *f* = 100 mm focal length telecentric f-theta lens. Such a configuration resulted in the laser beam waist at the focal plane of $\omega _0$ = 21 $\mu$m (at $1/e^2$ level), which was determined using the Liu method^33^. The positioning of the sample in all three (*x*,*y*,*z*) directions was achieved using high-precision nanopositioning stages (“ABL1500”, Aerotech, Inc.). Due to the small size of the ablated structures, all laser processing experiments could be conducted within the field of the f-theta lens (which covered an area of 50x50 mm).

**Fig. 9:**
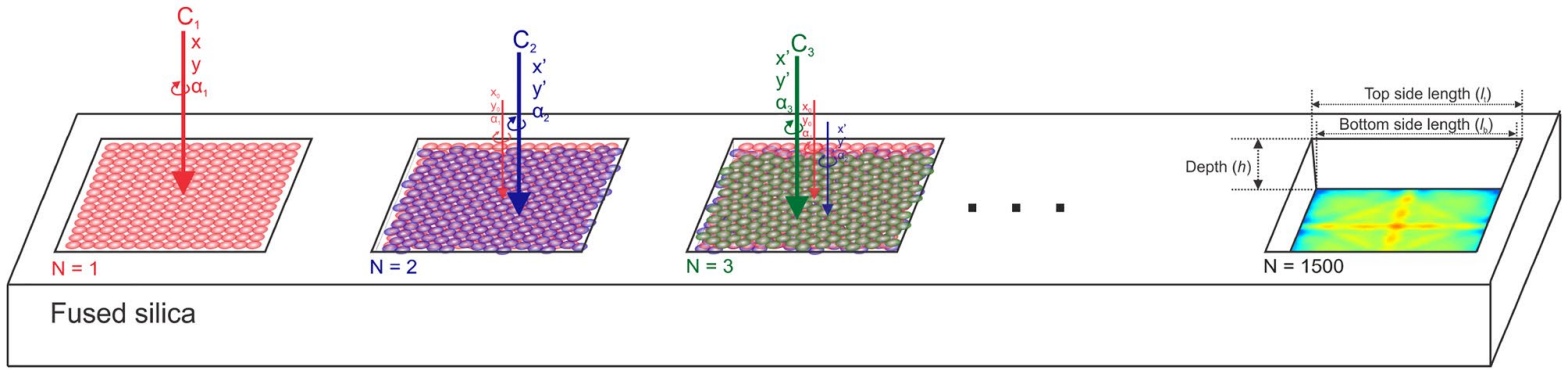
Schematic of the laser engraving process and scanning strategies. Arrows, denoted as $c_1$, $c_2$ and $c_3$ display the central axis of each layer with corresponding spatial coordinates *x*, *y* and angle of rotation $\alpha _n$. Different colors stand for different layers. *N* stands for number of scans performed on the surface.

The experiments consisted of scanning the surface area of 4x4 mm (or similar) in size multiple times, until the desire depth was reached. During laser processing, the focal plane was maintained at the sample surface. The Rayleigh length, calculated as $z_r$ = 1.35 mm, was sufficiently long to sustain efficient ablation across the entire deep engraving procedure, eliminating the need to adjust the focus between the scans. The scanning was performed in a raster manner, line by line, with a line pitch of *dy*. To maintain even ablation, the overlap of the craters in *x* and *y* directions was kept equal and was expressed as:

3 $$\begin{aligned} \Omega _i = \left( 1 - \frac{di}{D}\right) \times 100\%, \quad i \in \{x, y\}, \end{aligned}$$

here $\Omega _{x,y}$ - the overlap of craters in *x* and *y* directions, $dx =dy$ - the distance between adjacent craters in *x* and *y* directions, *D* - the diameter of a single crater. The overlap in the *x* direction was varied by changing the scanning speed (*v*), while the overlap in the *y* direction was varied by selecting different line pitch values (*dy*). Furthermore, when scanning multiple times, to avoid the formation of the periodic structures that arise from the raster scanning, additional scanning strategies - rotation of the layers and the displacement of the layers were implemented. The visual representation of laser engraving employing the aforementioned scanning strategies is shown in Fig. 9. When layer rotation is applied, each successive layer is rotated about its central axis by a fixed incremental angle $\alpha$. In addition, layer displacement involves shifting the central rotation axis of the current layer by a randomly generated offset in the *x* and *y* directions, thereby further reducing the likelihood of laser irradiation at the same locations. The presented scanning strategies were found to be most effective in achieving uniform ablation of dielectric materials, as detailed in^14^. The surface scanning, incorporating all the previously described strategies, was carried out using DMC PRO (Direct Machining Control, UAB) software.

After laser processing, the engraved structures were subjected to the cleaning procedure which included cleaning the samples in an ultrasonic bath filled with distilled water for the duration of > 15 min and subsequently drying them using high-pressure air. Next, the engraved surfaces were measured using the LEXT OLS5100 laser scanning microscope (Olympus Corporation) equipped with a long working distance 50$\times$ objective (LMPlanFLN 50$\times$, Olympus) with NA = 0.6. This configuration provided a lateral resolution of $r_l \approx 0.25\,\mu$m and a diffraction-limited axial resolution of $r_a \approx 1.5\,\mu$m. Despite this optical axial limit, the system could achieve an effective height measurement precision on the order of $\sim 10\,\text {nm}$ through high-accuracy peak detection and intensity-based surface fitting of the confocal signal. Due to the limited single field of view (256 $\times$ 256 $\mu$m), multiple adjacent measurements were stitched together to capture the entire engraved surface. The acquired surface topographies were digitally processed to remove measurement noise and the form of the surface, which does not contribute to the surface roughness. Next, 3D surface roughness parameter $S_q$ was calculated to evaluate surface roughness level. It represents the root mean square value of ordinate values within the definition area and expresses as^34^:

4 $$\begin{aligned} S_q = \sqrt{\frac{1}{A} \iint _{\tilde{A}} |z^2(x,y)|dxdy}, \end{aligned}$$

here $S_q$ is the RMS roughness, $\tilde{A}$ represents the domain of integration, *A* - the value of the evaluation area, and $z^2(x,y)$ is the normal distance from the reference surface squared. This parameter was chosen for the evaluation of the surface roughness as it gives a good approximation of general roughness value and is widely used within the literature. Note that $S_q$ values here were calculated for SF surfaces^35^, which include both roughness and waviness parts of the surface texture. The decision to compute $S_q$ for SF surface, rather than the SL surface, is motivated by the importance of evaluating the influence of induced high-amplitude low-spatial-frequency features on the overall surface unevenness.

## Data Availability

Data underlying the results presented in this paper are not publicly available at this time but may be obtained from the authors upon reasonable request.
